# A curation system of rice trait ontology with reliable interoperation by LLM and PubAnnotation

**DOI:** 10.1186/s44342-025-00058-z

**Published:** 2025-12-02

**Authors:** Javeed Muhammad Ahmad, Yawen Liu, Jin-Dong Kim, Xinzhi Yao, Pierre Larmande, Jingbo Xia

**Affiliations:** 1 College of Informatics, Hubei Key Laboratory of Agricultural Bioinformatics, Huazhong Agricultural University, Wuhan, China; 2 Database Center of Life Science, Tokyo, Japan; 3 French National Research Institute for Sustainable Development (IRD), Marseille, France; 4 University of Montpellier, Montpellier, France

**Keywords:** *Oryza Sativa*, Rice-Alterome, Large language model, Rice trait ontology, Trait curation system, PubAnnotation

## Abstract

**Background:**

Ontology frameworks are essential for organizing complex biological knowledge, such as genes, phenotypes, and pathways, and for ensuring consistent data annotation and retrieval. In biological research, ontologies like the Gene Ontology (GO) and crop-specific trait ontologies (TO) for *Oryza sativa* (rice) standardize terminology across studies, supporting cross-study comparison and hypothesis generation. However, ontology annotations usually rely on expert manual review of the literature, a process that is accurate but time-consuming, labor-intensive, and difficult to scale as biological data grows. Manual approaches are also prone to inconsistencies and errors. The emergence of large language models (LLMs) such as ChatGPT, DeepSeek, and KIMI, along with curated databases like Rice-Alterome and PubAnnotation, offers new opportunities for semi-automated ontology curation. This study explores how these technologies can be integrated to develop an efficient literature-based curation system for rice trait ontology.

**Methods:**

We developed a curation system that integrates Rice-Alterome—a comprehensive database of rice genomic variations, mutations, and sentence-level literature evidence linked to GO and TO terms with PubAnnotation, an open-source platform for collaborative text annotation. LLMs (DeepSeek and KIMI) were integrated via APIs to automate the extraction, annotation, and validation of trait-related information via prompt engineering. The system was evaluated through use cases designed to demonstrate its performance and functionality compared to manual curation.

**Results:**

The proposed system substantially enhanced the retrieval and organization of literature evidence compared to manual methods. The integrated platform, available through a dedicated website, connects Rice-Alterome, PubAnnotation, and LLMs to streamline ontology curation and evidence discovery. This framework reduces the time domain experts need to locate and validate relevant information and provides interactive tools for users to add, merge, or refine trait annotations. The LLM-driven prompt-based querying also improved the identification of implicit or missing information that may be overlooked during manual curation.

**Conclusions:**

Integrating LLMs with Rice-Alterome and PubAnnotation offers a promising solution for automating rice trait ontology curation. This approach accelerates evidence collection and enhances data consistency and accessibility. Future extensions of this framework will target additional crops such as wheat and maize and focus on refining LLM-based retrieval and annotation mechanisms for broader agricultural genomics applications.

## Introduction

Rice is a staple crop whose demand and production are increasing daily. At the same time, new genetic and genome information emerges. Reviewing large volumes of biological literature and extracting reliable data from authentic sources is a central challenge in biological research [1, 2]. Multiple data centers, such as PubMed [3], Oryzabase [4], and Planteome [5], provide trait ontology verification and evidence collection for specific entities. For experts, searching a vast amount of data related to rice trait ontology (RTO) [6] can be a difficult and overwhelming task. They often struggle to interact with many tools to obtain accurate definitions of trait ontology, especially in specialized fields. Many trait ontologies share similar definitions across different domains, causing confusion and ambiguity when trying to establish clear meanings. Ontologies are typically hierarchical, with multiple levels of parent-child relationships. Navigating this complexity to find the exact term that matches a specific experimental condition or biological context can be challenging.

**Fig. 1:**
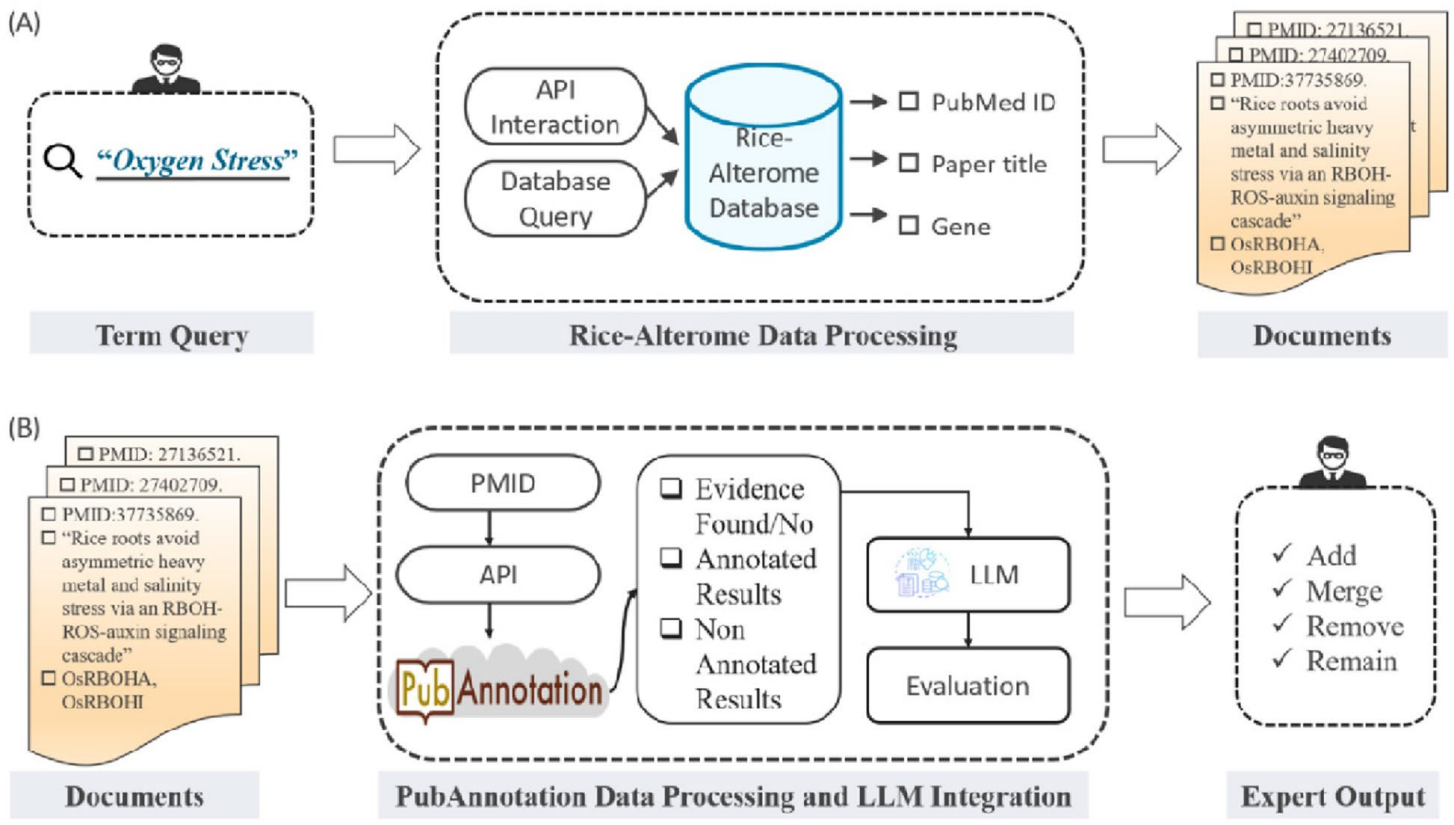
Workflow of the project. **A** Integration of Rice-Alterome and document retrieval which includes the Gene, Phenotype, and Sentence to explain their trait. **B** Retrieved documents will be used to collect evidence from PubAnnotation through PubAnnotation APIs. Leverage the functionality of LLM and enhance the reliability of evidence. Results obtained from Rice-Alterome integration need to be processed through PubAnnotation reflecting the evidence results and letting experts decide with further options

With the leverage of new technologies such as Natural Language Processing (NLP) [7] in biological research, these challenges can be addressed. Using various deep learning models [8], such as Transformers [9] and BERT [10], helps resolve terminology based on biological knowledge. However, some issues may arise: (i) training these diverse models requires data from scratch; (ii) traditional NLP often involves multi-level processing, which can lead to complications; (iii) limited biological resources can hinder the training process. Genome-wide association studies (GWAS) also play a central role in providing gene-trait associations, offering concrete information about the relationship between genes and traits, though they are generally used across different species and are not specific to rice, nor do they provide literature evidence [11]. The IRRI rice Trait Development Pipeline describes trait development across several biological aspects and ensures the reliability of genes and markers [12]. Gramene [13], a useful tool specifically developed for trait systems and vocabulary related to plant anatomy, relies on manual work to develop literature evidence, making it a time-consuming task for experts. To address these issues and reduce human involvement, our project in BLAH9 focuses on integrating Rice-Alterome and PubAnnotation with large language models (LLMs) [14]. Rice-Alterome is a curated dataset that includes multiple annotations associated with PubMed IDs, such as genes, mutations, variations, phenotypes, EnterzIDs, GO terms, and TO terms. PubAnnotation is an open-source, scalable, and shareable storage system for literature annotations that can be integrated with various systems [15, 16]. Recently, advances in LLMs have demonstrated remarkable capabilities in natural language understanding and generation. LLMs can connect with external tools via REST APIs to enhance their capabilities. For example, the KIMI moonshot [17] and DeepSeek [18] models are used in this research for rice literature analysis to provide knowledge-based, concrete evidence, helping researchers gather information on specific terms. However, LLMs may sometimes produce factually inaccurate or hallucinatory outputs, posing a challenge to academic rigor. To mitigate hallucinations, a controlled knowledge environment containing Rice-Alterome and PubAnnotation is employed. As a result, knowledge-based and reliable results can be obtained (Fig. 1). Through evaluation, we found that LLM responses outperformed those of other generative models due to the concrete knowledge bases from Rice-Alterome and PubAnnotation. We also highlight concerns regarding LLM applications, including challenges in low-resource domains and the associated computational costs.

To evaluate the system, we conducted a manual assessment using Fleiss’ kappa (kkk). Expert annotators labeled the sentences, and these labels were used to calculate the kappa score, yielding a value of 0.719. Based on these results, the curated system shows reliable performance and can be considered appropriate for aggregating collective evidence.

## Materials and methods

In this section, experimental results are presented on data set interoperability across multiple systems, conducted during the BLAH9.

### Databases

#### Rice-Alterome

Rice-Alterome is a curated data set designed by the HZAU-BioNLP team. This dataset contains more than 500 k sentences, which contain the relationship between genes and named entity relationships (NER). Moreover, Rice-Alterome is directly linked to PubMed literature IDs, which provide evidence for a particular sentence. Each sentence is properly annotated with different terms, such as gene, mutation, effects, stress, and others. The dataset is organized by gene, comprising approximately 8169 genes. For each gene entry, the dataset provides associated descriptive sentences that characterize gene functions and properties, along with annotated GO terms (e.g., “carbon fixation” and “response to oxygen stress”) and TO terms accompanied by their respective definitions. The Rice-Alterome system provides a web service with a RESTful API, built upon the RTO—an ontology that hierarchically organizes key traits and their parent-child relationships. This open-source platform enables researchers to access annotated data, including GO terms programmatically, TO terms, gene-mutation associations, and named entity recognition (NER) results from the literature. Experts can integrate this API to extract structured information for downstream analyses. The process illustrated in Fig. 1A describes the integration between our experts and the Rice-Alterome database, demonstrating how users can query the dataset and how the curation system performs back-end operations responsively while extracting relevant information. The information provided by the database will include the PMID of the literature from PubMed, sentences associated with the literature, and terms mentioned by the user. This service can be customized to offer further information, for example, the release time of this evidence and its reliability. This specific integration is written in Python Django, and the extracted results are in JSON format.

#### PubAnnotation

PubAnnotation is an open source repository that consists of various database collections uploaded by different contributors. It is composed of powerful algorithms and logics that help the user upload their own database, annotate it automatically and align it with the data existing in PubAnnotation. Thus, PubAnnotation eliminates the need for manual annotation, significantly reducing expert workload and improving time efficiency.

In this research, PubAnnotation is integrated to support the extraction of concrete knowledge from Rice-Alterome. PubAnnotation provides storage, alignment, titles, abstracts, annotations, named entity recognition and evidence-based annotations based on PubMed ID [19]. Here, we describe how our system is integrated with PubAnnotation in Fig. 1. As explained in the above Fig. 1, it gets the source data from Rice-Alterome and maps the evidence data with PubAnnotation. PubAnnotation is exploited through the REST API, which is a flexible and reliable option. With the help of PubAnnotation, domain-specific knowledge focused on rice-related data can be obtained.

#### Data integration with Rice-Alterome and PubAnnotation

Integrating Rice-Alterome and PubAnnotation within the curation system is crucial for improving the understanding of rice trait ontology. Rice-Alterome provides comprehensive details on rice, including gene information, PMID, alterations, slicing, and mutations, whereas PubAnnotation provides standardized annotations of biological entities in the OryzaGP database [20]. By merging these datasets into a curation system, researchers can standardize and connect varied data sources using a unified framework. This integration not only promotes consistency and interoperability but also enables intricate analyses and the identification of rice traits. Ultimately, this cohesive approach enhances interoperability and enables researchers to deliver valuable knowledge support. The Rice Trait Ontology dataset includes approximately 2051 traits in detail, including their definitions, synonyms, parent-child relations among these traits, and so on. This dataset can be accessed and integrated via an API. For instance, for the trait “carbon sensitivity”, results obtained from Rice-Alterome include the PMID, gene, sentences, GO term, and TO term. After analyzing the results from Rice-Alterome, researchers can filter the outcomes to identify the most relevant information. These results will then be integrated with PubAnnotation to provide supportive literature with annotations. The integration will use an API to facilitate rapid information extraction, as illustrated in Fig. 1.

### Development of intelligent system for rice ontology curation

#### Main functions of the curation system

The RTO curation system enables rice trait ontology experts to evaluate and classify traits through an interactive web interface. Experts can make curation decisions by selecting one of the following actions: (i) ADD a new class; (ii) MERGE a term into an existing trait; (iii) REMOVE the existing class/trait; (iv) REMAIN as it is. This will help the expert to form the decision regarding the existing trait or new class. This project is handled both from front and back-end where each term or trait will be integrated with Rice-Alterome and PubAnnotation through REST API. The system architecture employs a Python Django back-end framework coupled with a React-based front-end, integrated as a single application. Django, a robust and scalable web framework, facilitates both server-side operations and front-end rendering while demonstrating efficient handling of large-scale datasets.

Once an expert searches for or examines a term in the rice trait ontology, they first collect evidence from various literature sources like Rice-Alterome, as shown in Fig. 1A, and PubAnnotation, as shown in Fig. 1B. The collection of literature and knowledge support will help the expert make decisions related to the different functions mentioned earlier. If literature evidence is not sufficient from the resources, the LLM provides assistance due to its reliability. In this case, other literature data from external resources can be presented by the LLM, which will aid in decision-making.

#### Automated web services

The integration of large-scale, complex systems such as Rice-Alterome and PubAnnotation poses a significant challenge, often requiring substantial time and effort to map and connect their primary data sources. A critical simplifying factor is that both systems use the PubMed ID (PMID) as a common identifier. This provides a robust foundation for a unified data retrieval strategy.

To circumvent the inefficiency of manually gathering runtime evidence for user queries—a highly time-intensive task—we propose an integrated web services framework. This framework automatically queries both systems concurrently, presenting consolidated results to the user and thereby preserving expert resources for evaluation rather than data collection.

This architecture also extends to the handling of novel traits. Upon the discovery of a term absent from the system, an automated pipeline can be initiated. The term undergoes a team-based validation procedure, which includes curating supporting literature and assessing information from established databases. Once validated and integrated into the ontological tree, the automated services immediately incorporate the new term, enabling seamless data acquisition and maintaining the system’s currency.

#### Experts’ interaction with the curation system

The web interface is designed to facilitate expert-driven curation, enabling critical knowledge management functions such as adding, merging, retaining, and removing ontological terms. This interaction is central to advancing the system’s capabilities, paving the way for automated term discovery and evidence collection from literature to construct a robust, evidence-based knowledge graph.

The user journey is structured into distinct tiers of functionality. Initially, any user can search and explore the existing ontology. Upon verification and authentication, experts are granted access to an advanced suite of curation tools. This allows them to formally submit requests for ontological changes—such as proposing a new trait, merging duplicates, removing obsolete terms, or retaining existing ones.

Each submitted request undergoes a validation process to ensure data integrity and ontological consistency. Once a request is validated and approved, the proposed changes are integrated into the live curation system, thereby continuously refining and expanding the knowledge base through a controlled, expert-driven workflow.

#### Advanced service with LLM integration

Leveraging tools like large language models has made ontology development more efficient and accurate. LLMs generate concise, context-rich definitions for ontology terms based on existing literature. This structured approach creates a clear relationship between traits, definitions, and relevant literature. For human experts, these tools provide comprehensive definitions and literature annotations, requiring only verification to ensure reliability, ultimately enhancing the accuracy and robustness of the ontology as a trustworthy resource for researchers.

Integration with the LLM can be achieved in various ways, such as through an API or a website. Currently, many systems have enhanced their capabilities through LLM and the most common and efficient access to the LLM is through API. However, interacting with the LLM also leads to the fabrication of the results due to hallucination. To avoid this, we conducted multiple tests to obtain feasible results from the LLM. The final prompt will be saved in the backend, and it will be modified according to the user terms entered. Figure 1B showcases the interaction flow with the LLM and then displays the results to users. If the expert encounters the same trait, the relevant results can be found without any further LLM query searches, as an expert can see both prompts and results from LLM (DeepSeek, KIMI), as previous experts made, which makes the decision-making easy.

The web page integrated KIMI and DeepSeek to provide concrete knowledge that can support the existing knowledge. Users need to provide their API keys to access the relevant responses from these LLMs.

#### The web application

Interacting with the curation system is a vital aspect of collaboration among various experts. Experts visit the site to check the definitions of traits, synonyms, and their relationships with other traits. Notably, users can search for specific traits using entities, determine whether a given entity exists, and evaluate it based on relationships and comments from experts. If they wish to add a trait, they can easily select it with just a click. However, the purpose of the curation system extends beyond this: integrating PubAnnotation with Rice-Alterome can enhance the collection of evidence related to traits.

The web application tool offers the following functions: (i) searching for terms/traits to verify existing entries in the hierarchical tree; (ii) integrating Rice-Alterome and PubAnnotation datasets to gather evidence; (iii) integrating with LLMs to enhance comprehensive results; (iv) evaluating traits and facilitating the addition or merging of traits based on expert opinions. Python and Django are used for backend tasks and data services, while the graphical user interface (GUI) is developed with ReactJS. By employing advanced strategies, we can save time and improve code usability. Finally, our web platform will provide interactive visualization with the following key features: (i) user-friendly query interfaces, enabling searches based on both custom terms and the rice trait ontology (RTO), alongside core analytical functions; (ii) a feedback mechanism to integrate user input, as the current limitations of datasets, search scenarios, and large language model (LLM) complexities necessitate human expertise for optimal performance. Concurrently, we are optimizing the underlying data structure to ensure responsiveness and interactivity.

## Results

In this section, various use cases were explored, which can assist experts in determining whether the term they searched needs to be added, merged, removed, or retained as they are. The following use cases illustrate the main functionality of the curation system.

### Use cases

#### Use case 1: addition of a new class

Adding a new Trait to the trait ontology is a brainstorming process that involves gathering substantial evidence from diverse resources, making it challenging to decide whether it constitutes a valid new trait or an invalid one. An example is presented which includes the trait name, the amount of evidence, the resources of the evidence, and the reasons for its inclusion as a class within the trait ontology system. The trait name is “Seedling-stage submergence tolerance” in rice, which does not currently exist in the rice trait ontology and represents early-stage water stress during seed germination. Evidence is derived from Rice-Alterome, PubAnnotation, and NCBI, totaling over 100 results.

The following texts support these pieces of evidence:

i) “Biocuration of a Transcription Factors Network Involved in Submergence Tolerance during Seed Germination and Coleoptile Elongation in Rice (Oryza Sativa)” [21].
ii) “Flood resilience loci SUBMERGENCE 1 and ANAEROBIC GERMINATION 1 interact in seedlings established underwater” [22].
iii) “Elongated coleoptile contributes to submergence tolerance during germination of direct seeded rice. Here, the authors show that natural variation of rice coleoptile length is determined by the glycosyltransferase encoding gene OsUGT75A by reducing free ABA and JA levels through glycosylation of these two phytohormones” [23].

These references portray it as a trait and link it to submergence tolerance, which is already included in the rice trait ontology. Therefore, we can add a new class under submergence tolerance, designating the trait “Seedling-stage submergence tolerance” as a child of the parent trait called submergence tolerance which can be visualized in hierarchical structure.

#### Use case 2: merging of one class into another class

Another important function for an RTO expert is the intensive effort needed to merge an existing term or trait in the rice trait ontology. In the current version of the rice trait ontology, there are two similar traits, “submergence tolerance” and “flooding tolerance.” Both of them belong to the same parent trait named “submergence sensitivity”. Flooding tolerance closely resembles submergence tolerance and can be viewed as a synonym for submergence tolerance. We can consider the trait example of submergence sensitivity, which relates to the water tolerance trait. One of the traits named “submergence tolerance,” which most closely resembles “flooding tolerance,” acts as a synonym for submergence tolerance. Evidence can be found in both Rice-Alterome and PubAnnotation, reflecting results related to flood tolerance in the rice crop. From the Rice-Alterome dataset, it shows 39 sentences and PMIDs, as well as the same number of genes associated with the submergence tolerance and flooding tolerance traits, which we can conclude in the following sentences;

i) “In most wild-type rice cultivars, which are not equipped with the submergence tolerance regulator SUBA1 or SNORKEL1 and SNORKEL2 in their genotypes, the rapid increase in OsETR2 expression may become a more important protective and adaptive measure when they encounter flooding and submergence” [24].
ii) “The elongation ability reduced with consequently greater submergence tolerance when floodwater increases rapidly resulting in complete submergence, as often occurs in flash-flooding” [25].
iii) “Allelic sequence variation in the Sub1A, Sub1B and Sub1C genes among diverse rice cultivars and its association with submergence tolerance Erratic rainfall leading to flash flooding causes huge yield losses in lowland rice” [26].

As reflected in the references, it is feasible to consider that flooding tolerance is the synonym of submergence tolerance which can be merged into submergence tolerance.

#### Use case 3: removal of a class

Removing a trait from the ontology system is a daunting task. A trait may be removed for the following reasons: (i) insufficient evidence found in the literature, resulting in experts wanting to retain it for future research, while sometimes preferring to remove it; (ii) a trait that is considered a duplicate when multiple entries represent the same concept. Despite these considerations, unfortunately, the current rice trait ontology system does not contain any false traits that can be removed, whether they are duplicates or have inadequate literature evidence.

#### Use case 4: population of a class with evidences

While working with the rice trait ontology, we may find many traits that already exist, and there is sufficient literature evidence associated with existing traits. Here, we provide the example of “submergence tolerance” and “Cadmium toxicity,” which already exist in Rice-Alterome, with evidence counts of 207 and 50, respectively. The retrieved dataset has also been verified by PubAnnotation, confirming the evidence of their existence. The following sentences represent the evidence of trait existence in the literature for “Cadmium toxicity.”

i) “Alleviation of cadmium toxicity in Zea mays L. through up-regulation of growth, antioxidant defense system and organic osmolytes under calcium supplementation Calcium (Ca) is a macronutrient and works as a modulator to mitigate oxidative stress induced by heavy metals.” [27].
ii) “Mitigating effects of the exogenous application of SPD, SPM, and PUT during heavy metal induced stress were reported in wheat exposed to increased lead and cadmium levels, which resulted in beneficial effects of polyamines, increased plant tolerance to heavy metals, and reduced metal phytotoxicity.” [28].
iii) “Effect of potassium deficiency on antioxidant status and cadmium toxicity in rice seedlings Cadmium (Cd) is one of the most toxic heavy metals and inhibits physiological processes of plants” [29].

Taking into account that there is sufficient evidence to support “Cadmium toxicity” from the literature, the user concludes to retain it as it is in RTO.

### Web application GUI

**Fig. 2:**
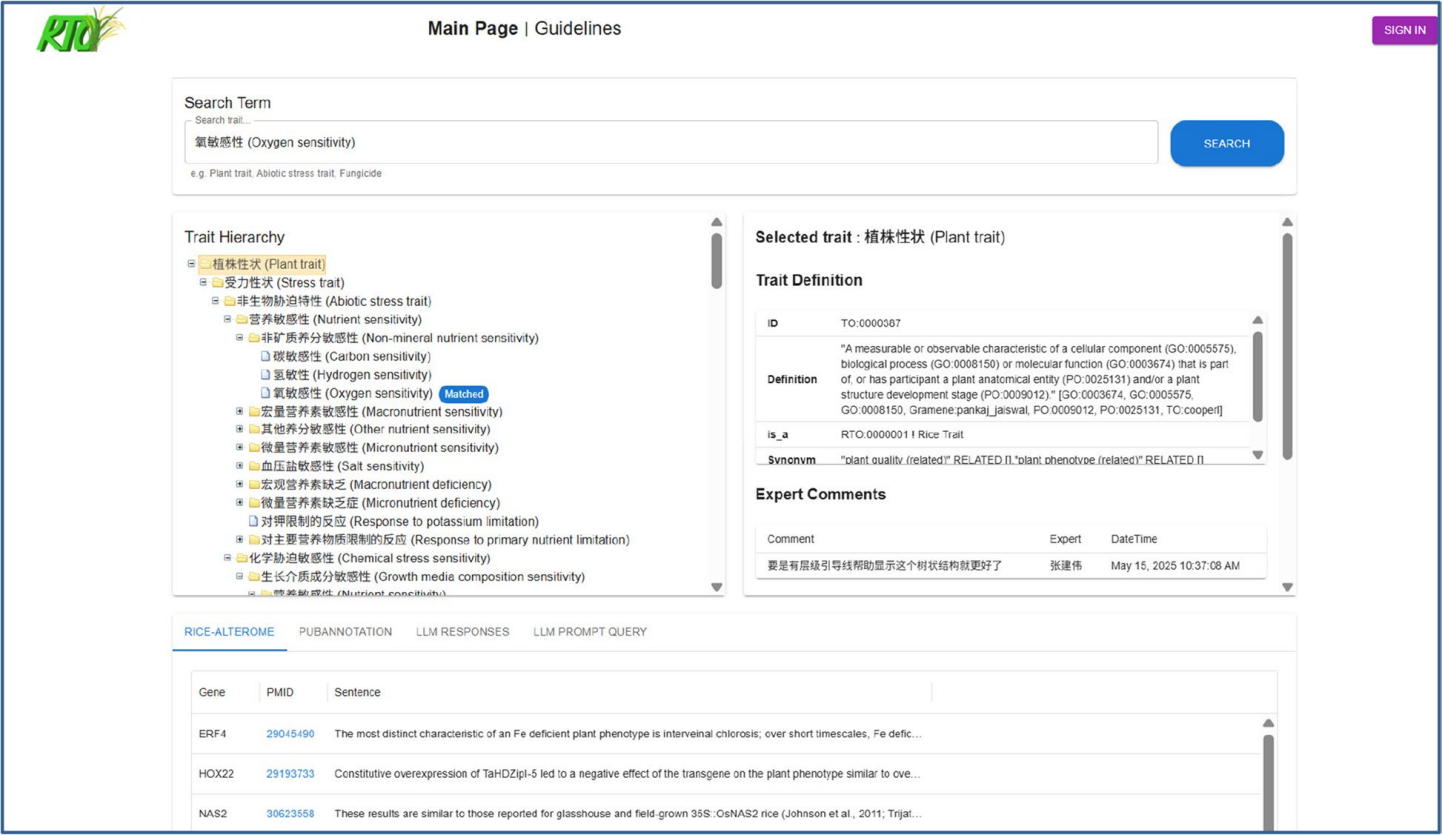
The layout of the rice trait curation system webpage. The webpage is divided into four components: (i) search trait from tree and match tags; (ii) hierarchical relation tree; (iii) definition and expert comments, and actions performed by experts; (iv) the bottom is the evidence related to the selected trait from Rice-Alterome and Pubannotation

The webpage is designed to support experts by providing access to a search form and allowing them to perform specific actions related to the trait. Evaluation actions can be performed without a login, but other term-processing actions, such as add, merge, remain, and remove, require a user’s login. The webpage is divided into four components: (i) trait search with auto completion trait dropdown list; (ii) trait hierarchy to check the relation between other traits and terms, which provides experts with further details related to the selected trait; (iii) when a trait is selected, the curation system retrieves the selected trait details (ID, definition, and synonyms); (iv) selecting a trait allows experts to see the supporting evidence table from Rice-Alterome, PubAnnotation, LLM response through prompt engineering, and gives the right to experts to query from LLM (KIMI and Deepseek) from a selective prompt that can be modified. The web page (Fig. 2) includes two sections: Guidelines and Curation system. The guidelines section includes important information related to the curation system. For more details, please check the published webpage Rice Trait curation system (http://lit-evi.hzau.edu.cn/rice_trait_ontology_curation_system/).

### Evaluation of curation system

**Fig. 3:**
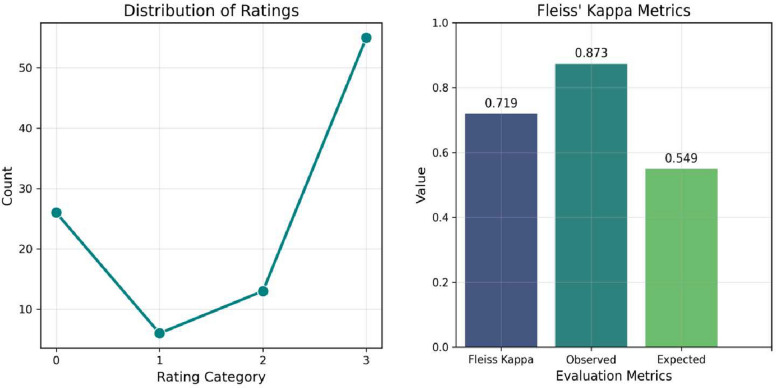
Distribution of rating categories (0–3) across raters (line graph) and inter-rater reliability metrics including Fleiss’ kappa, observed agreement, and expected agreement (bar graph). The results show a higher concentration of ratings in category 3 and substantial agreement among raters (*κ* = 0.719)

We manually evaluated the curation system’s outputs by distributing results for approximately 20 traits among three domain experts. Each expert independently classified the sentences as either positive (1) or negative (0) for the respective traits. Following the classification, we aggregated the responses from the three experts by summing their individual scores for each sentence. The resulting values, illustrated in Fig. 3, represent the cumulative classification outcomes across all 20 trait-related sentences. Figure 3 further visualizes these aggregated results, providing a comprehensive overview of the experts’ consensus. To quantitatively assess the reliability and consistency of the curation system, we employed Fleiss’ kappa, a statistical measure of inter-rater agreement. As depicted in Fig. 3**,** the calculated kappa value allows us to estimate the validity and robustness of the system, indicating the extent to which the expert evaluations align and confirming the reproducibility of the classification process.

## Discussion and conclusion

This study demonstrates the integration of two specialized databases with a large language model (LLM) to help experts generate accurate and reliable conclusions. The system helps users assess the relevance of proposed rice trait ontology terms. It includes expert authentication for secure login and key trait operations like addition, merging, removal, and population. Without authentication, experts can only retrieve existing evidence of selective traits. The curation platform ensures only authenticated experts can perform these actions, allowing them to submit annotations on selected terms. Users can also retrieve term definitions and submit supportive evidence through a unified interface, consolidating relevant data for analysis. We explore the utility of LLMs by extracting rice-related knowledge from both literature and LLM-generated outputs. For LLM-based evidence, we designed prompts that include traits, allowing the LLM to retrieve relevant information from the literature. Both literature-based and LLM-based evidence were evaluated manually to assess accuracy, including checking for unsupported or hallucinated content from the LLM.

Experts play an important role in this system. If the term does not exist in the curation system, an expert can add and merge the new term within the system. After evidence collection and evaluation, the new term can become public. This curation system helps experts save time by quickly finding existing evidence in the literature. Our curation system matches terms using strict and fuzzy matching to collect evidence. We have about 2051 existing traits integrated with Rice-Alterome and PubAnnotation. If evidence is not found, experts can query the LLM resource by applying their API key. This enhances the expert’s ability to make decisions about the curation system’s functionality.

The distribution of ratings shows that category 3 was most frequently assigned (55 instances), followed by category 0 (26), category 2 (13), and category 1 (6). This suggests raters generally favored higher ratings. This pattern may reflect the true prevalence of the measured trait or a tendency toward higher scoring. The calculated Fleiss’ kappa value is 0.719, indicating substantial agreement among raters beyond chance (expected agreement = 0.5490). The high observed agreement (0.8733) shows raters were largely consistent in their assessments. These results demonstrate the rating process is reliable. The slight difference between observed and expected agreement highlights minor variations in rater judgment, which might be addressed with additional guidelines or calibration. The analysis shows substantial inter-rater reliability for this dataset, with a Fleiss’ kappa of 0.719. This suggests the ratings are generally consistent and reproducible, supporting the validity of the categorization process. While minor discrepancies exist, the overall agreement is strong enough to provide confidence in the reliability of the raters’ evaluations.

## Data Availability

All code and data are available on the GitHub project, https://github.com/Ahmad4321/rice-trait-curation-system.
